# Data-Driven Analysis of Fluorination of Ligands of Aminergic G Protein Coupled Receptors

**DOI:** 10.3390/biom11111647

**Published:** 2021-11-08

**Authors:** Wojciech Pietruś, Rafał Kurczab, Dagmar Stumpfe, Andrzej J. Bojarski, Jürgen Bajorath

**Affiliations:** 1 Department of Medicinal Chemistry, Maj Institute of Pharmacology, Polish Academy of Sciences, Smetna 12, 31-343 Krakow, Poland; pietrus@if-pan.krakow.pl (W.P.); bojarski@if-pan.krakow.pl (A.J.B.); 2 Department of Life Science Informatics, LIMES Program Unit Chemical Biology and Medicinal Chemistry, B-IT, Rheinische Friedrich-Wilhelms-Universität, Friedrich-Hirzebruch-Allee 6, D-53115 Bonn, Germany; stumpfe@bit.uni-bonn.de

**Keywords:** G protein-coupled receptors, GPCR, aminergic receptors, fluorine, activity cliffs, MMP, ChEMBL

## Abstract

Currently, G protein-coupled receptors are the targets with the highest number of drugs in many therapeutic areas. Fluorination has become a common strategy in designing highly active biological compounds, as evidenced by the steadily increasing number of newly approved fluorine-containing drugs. Herein, we identified in the ChEMBL database and analysed 1554 target-based FSAR sets (non-fluorinated compounds and their fluorinated analogues) comprising 966 unique non-fluorinated and 2457 unique fluorinated compounds active against 33 different aminergic GPCRs. Although a relatively small number of activity cliffs (defined as a pair of structurally similar compounds showing significant differences of activity −ΔpPot > 1.7) was found in FSAR sets, it is clear that appropriately introduced fluorine can increase ligand potency more than 50-fold. The analysis of matched molecular pairs (MMPs) networks indicated that the fluorination of the aromatic ring showed no clear trend towards a positive or negative effect on affinity; however, a favourable site for a positive potency effect of fluorination was the ortho position. Fluorination of aliphatic fragments more often led to a decrease in biological activity. The results may constitute the rules of thumb for fluorination of aminergic receptor ligands and provide insights into the role of fluorine substitutions in medicinal chemistry.

## 1. Introduction

Currently, almost 700 unique human proteins are known drug targets, of which five of the most druggable target classes are: G protein-coupled receptors (GPCRs), ion channels, kinases, nuclear hormone receptors and proteases. GPCRs are a target for 34% of the global market share of therapeutic drugs [1,2], with aggregated sales for 2011–2015 of ~US$890 billion [3]. GPCRs are membrane-bound (located also in various intracellular compartments) receptors [4] used in the treatment of neurodegenerative, immunological, cardiac, and renal diseases, cancer [3], and many other disorders [5], as well as in many cases being important off-targets [6].

Selective fluorination of bioactive compounds is a well-established strategy in the design of new drugs to increase pharmacological efficacy, biological half-life, and absorption. Additionally, the fluorine substituent may affect protein-ligand affinity and selectivity [7,8,9]. Indeed, a continuous increase in the number of approved fluorinated drugs or drug candidates that enter clinical trials are observed and it is worth emphasizing that fluorine-containing compounds constitute over 50% of blockbuster drugs [10]. Fluorinated drugs cover all therapeutic areas, exhibit broad structural diversity, and contain a variety of fluorinated moieties, despite the limited covalent connectivity of fluorine atoms compared to other heteroatoms [11,12].

Considering the therapeutic potential of GPCRs and fluorine introduction as one of the most frequently used modifications of a lead compound to further improve its biological activity, we examined fluorine substituents in the context of activity cliffs (ACs). An AC is generally defined as a pair of structurally similar compounds having a large difference in potency.

First, we systematically searched for fluorinated aminergic GPCR ligands in ChEMBL. Ligand pairs and sets of pairs that differed only by fluorine atoms were identified and potency differences between fluorinated and non-fluorinated compounds were determined. In addition, substructure relationships between ligands pairs and sets were analyzed relative to underlying structure-activity relationships (SARs). The AC formalism was modified and applied to the fluorinated and non-fluorinated compound pairs.

Nearly 1200 non-fluorinated active ChEMBL compounds were identified across more than 35 qualifying GPCR targets that had at least one structural analogue containing one or more F atoms. For those ligand sets, a new network data structure was designed to investigate contributions of individual substitutions of F atoms to potency changes. The introduced network represents structural relationships between sets of compounds instead of individual compounds only. The non-fluorinated and fluorinated ligand sets are informative test cases for SAR exploration in light of the contributions of single or multiple fluorine atoms to the activity towards aminergic GPCR targets.

## 2. Materials, Methods, and Analysis Concepts

### 2.1. Compounds and Activity Data

Bioactive compounds were extracted from the ChEMBL database version 26 [13]. Only compounds with reported direct interactions (target relationship type: “D”) with annotation for 35 human aminergic GPCR [14] targets at the highest confidence level (target confidence score: 9), and exact measurements (“=”) were selected. In addition, only chosen potency measurements were taken into account (standard type: “K_i_”, “IC_50_”, “‘EC_50_”, “‘K_b_”, “K_d_”, “pK_i_”, “pIC_50_”, “pEC_50_”, “pK_b_”, “LogK_i_”, or “pK_d_”) and converted/reported as negative decadic logarithmic values. Given these criteria, a total of 21,800 unique compounds (44,033 measurements) with activity against 35 GPCRs were obtained and screened for the presence or absence of F atoms. Compound and activity data were extracted using in-house python scripts and KNIME (knime.org, accessed on 10 October 2021)protocols with the aid of Open Eye Toolkit (OpenEye Scietific Software, OEChem TK 2012).

### 2.2. Fluorine-Dependent Analogue Sets

**Figure 1:**
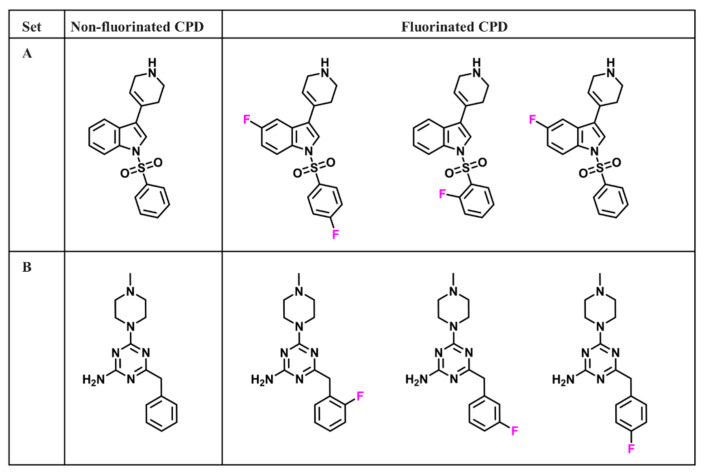
FSAR sets. Two exemplary FSAR sets A and B with ligands active against serotonin 6 (5-HT6) receptor are shown. On the left side, the non-fluorinated active compound of each set is shown and on the right side, corresponding active fluorinated analogues are shown.

The structures of all 21,800 compounds were systematically compared and if two or more compounds were active against the same target and differed only in the number of substituted fluorine atoms, requiring the presence of one non-F analogue, they were combined into an F-based analogue set for SAR analysis (FSAR set), as illustrated in Figure 1. Accordingly, 1554 target-based FSAR sets were identified, comprising 966 unique non-fluorinated and 2457 unique fluorinated compounds active against 33 different aminergic GPCRs (Table S1 in Supplementary Materials).

For each FSAR set, pairwise potency differences (ΔpPot) between the non-F compound and their fluorinated analogues were determined and categorized as no effect, positive effect, or negative effect (category (i), (ii), and (iii) below, respectively). Then, the union of all pairwise potency effects was generated and the effect for the FSAR set was assigned to category (i)–(v):

(i) No effect: MIN ΔpPot > −0.3 and MAX ΔpPot < 0.3
(ii) Positive effect: MIN ΔpPot ≥ −0.3 and MAX ΔpPot > 0.3
(iii) Negative effect: MAX ΔpPot ≤ −0.3 and MIN ΔpPot < 0.3
(iv) Mixed effect: MIN ΔpPot < −0.3 and MAX ΔpPot > 0.3
(v) Inconclusive effect: MIN ΔpPot ≤ −1.7 and MAX ΔpPot ≥ 1.7

**Figure 2:**
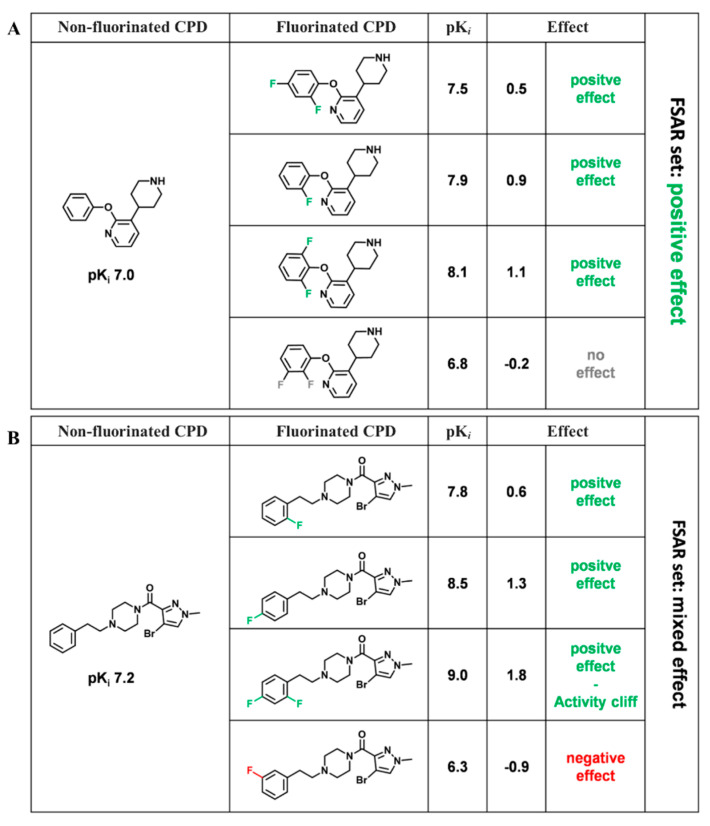
Potency effects. For two exemplary FSAR sets, the potency effect for each non-F (left)–fluorinated (middle) analogue pair and the resulting FSAR effects are given on the right. Potency values against serotonin 1a (5-HT1a) receptor (**A**) and serotonin 2a (5-HT2a) receptor (**B**) and ΔpPot are reported. As an AC criterion, a ΔpPot of 1.7 corresponding to a 50-fold difference in potency was applied.

The categorization is illustrated in Figure 2.

### 2.3. Activity Cliffs

Here, a pair of non-fluorinated/fluorinated analogues from the same FSAR set was classified as an AC if and ΔpPot was |1.7| or larger. A ΔpPot of 1.7 corresponds to a 50-fold difference in potency. As a standard criterion, AC formation often requires a 100-fold change in biological activity (i.e., ΔpPot > 2).

### 2.4. Matched Molecular Pairs

For a systematic similarity comparison of different FSAR sets, matched molecular pairs (MMPs) were calculated. MMPs were generated by systematic computational fragmentation of exocyclic single bonds in compound structures according to Hussain and Rea [15,16], and in this analysis, only transformation size-restricted MMPs [17] resulting from single-cut fragmentation [18] were sampled. A transformation size-restricted MMP is an MMP in which the shared core of the two molecules has at least twice the size of the exchanged substructures. In addition, the difference in size between the exchanged substructures is limited to at most eight heavy atoms, and both are not allowed to contain more than 13 heavy atoms [17]. For similarity assessment, MMPs were calculated for all 966 unique non-F compounds.

### 2.5. MMP Networks

**Figure 3:**
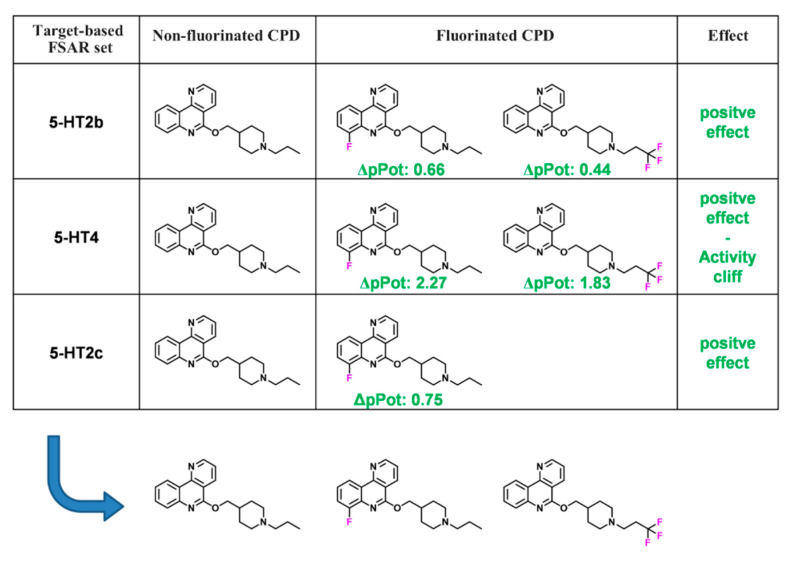
FSAR sets with multiple targets. Three target-based FSAR sets with ΔpPot values and their individual potency effects for the serotonin 2b (5-HT2b) receptor, serotonin 4 (5-HT4) receptor, and serotonin 2c (5-HT2c) receptor are shown (from top to bottom). For network analysis, target-based FSAR sets having the same non-F compound were combined into a “FSAR meta set” with a consensus potency effect.

MMP networks were generated in which nodes represented compound and edges pairwise MMP relationships. These networks were drawn with Cytoscape [19]. A non-F compound can be the origin of multiple FSAR sets with activity against different GPCR targets. Accordingly, the 1554 target-based FSAR sets contained 966 non-F compounds, a subset of which participated in multiple identical or overlapping target-based sets. For MMP network generation, these FSAR sets were combined, as shown in Figure 3, and might thus contain different potency effects for individual targets. The MMP network captured similarity relationships between the resulting 966 FSAR sets. In the MMP network, sets were color-coded according to the sum of all potency effects as follows:

(i). No effect: No effects for all targets (grey).
(ii). Positive effect: Only positive effects (possibly in combination with no effects) (green).
(iii). Negative effect: Only negative effects (possibly in combination with no effects) (red).
(iv). Mixed effect: Combinations of negative, positive, and mixed effects (possibly in combination with no effects) (yellow).

Each of the 966 FSAR sets was represented by its non-F compound as a single node and two nodes were connected by an edge if they formed an MMP. Nodes were color-coded by the corresponding effect(s) observed for the set (grey: no effect(s), green: positive effect(s), red: negative effect(s), yellow: mixed effect(s)). In addition, nodes were shown with a black border if at least one ΔpPot value within the set was larger or smaller than 1.7 and −1.7, respectively, thus representing an AC. If one non-F compound was a substructure of another and both formed an MMP (i.e., the MMP transformation involved a hydrogen atom in one compound and a non-hydrogen moiety in the other compound, the edge was colored in blue. All remaining edges were colored in grey.

## 3. Results and Discussion

### 3.1. FSAR Sets

From ChEMBL, all high-confidence GPCR ligands annotated with 35 aminergic GPCR targets were extracted and divided into two groups including non-F and fluorinated compounds. For each non-F compound, a search for fluorinated analogues was conducted, leading to the assembly of 1554 target-based FSAR sets with activity against 33 GPCRs, comprising a total 966 unique non-F GPCR ligands and 2457 unique fluorinated compounds. The size of the FSAR sets ranged from two to 13 compounds and 420 FSAR sets contained at least two fluorinated analogues. Based on the determination of pairwise compound potency differences and categorization of potency effects, as detailed in Materials and Methods, the 1554 target-based FSAR sets contained 617 sets with no potency effects, 387 with positive, 480 with negative, and 70 with both positive and negative effects. More than half (56%) of the FSAR sets were characterized by the presence of consistent potency effects for their target caused by the introduction of fluorine atoms. Only 4.5% of the detected potency effects were inconclusive and no FSAR set contained compound pairs with large opposing potency effects (ΔpPot ≥ 1.7). Hence, the majority of FSAR exhibited well-defined potency effects as a consequence of compound fluorination and provided a sound basis for SAR analysis.

### 3.2. Activity Cliffs in FSAR Sets and Their Interpretation

**Figure 4:**
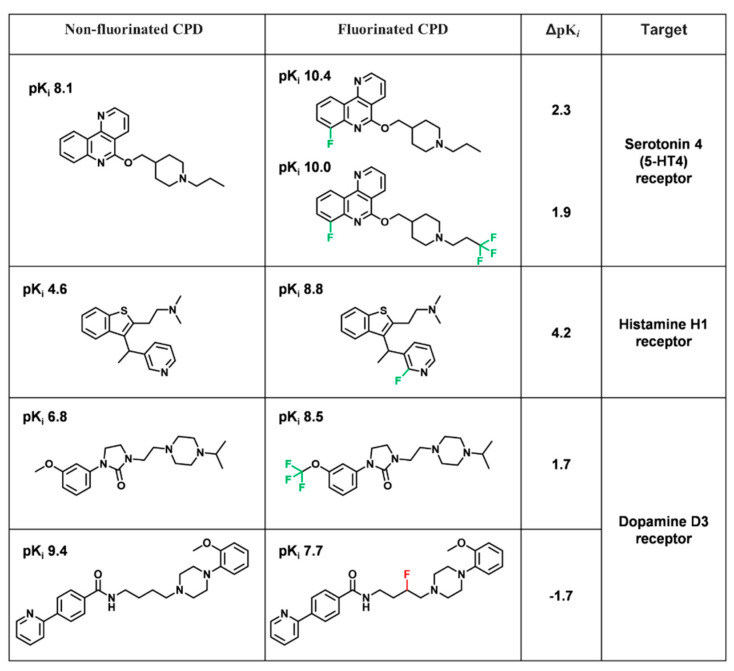
Exemplary activity cliffs. Five exemplary ACs resulting from compound fluorination are shown. For each non-F (left)–fluorinated (middle) compound pair, the ΔpPot (i.e., ΔpK*_i_*) value, and the corresponding target name are reported on the right. In addition, potency values for each compound are displayed.

The individual potency changes for compound pairs in FSAR sets ranged from ΔpPot of −2.8 to 4.2 with a mean of −0.06 (median −0.04). Hence, less than half of the compound pairs (~46%) had potency values within the expected range of measurement fluctuations. A subset of 1354 of the total 2526 compound pairs contained in the 1554 FSAR sets showed a ΔpPot of at least ±0.3 (i.e., a two-fold difference). However, only 54 compound pairs had a ΔpPot greater than 50-fold (±1.7) and were thus classified as ACs. In 48 (3%) of the FSAR sets, one to three ACs per set were identified for 18 different GPCRs. Hence, only 2.2% of all pairs represented ACs capturing an at least a 50-fold difference in potency. For comparison, the frequency of target-based ACs formed by analogue pairs in ChEMBL with at least a 100-fold difference in potency has remained essentially constant at ~5% of all pairs [20]. Accordingly, large-magnitude potency alterations as a consequence of compound fluorination were rare. Exemplary fluorination-dependent ACs are shown in Figure 4.

In the first example of 5-HT4 receptor ligands in Figure 4, the introduction of F atoms significantly improved the activity. However, molecular modeling studies indicated that in this case, fluorine did not form direct interactions in the binding pocket [21], suggesting that fluorine affected activity indirectly by, e.g., influencing the acidity/basicity and partial charges of adjacent atoms. Interestingly, the introduction of the trifluoro substituent changed the functional profile of the analogue from inverse agonist to antagonist [22].

Although fluorine is a bioisostere of hydrogen, the improvement of H1 receptor ligands activity in the second example might be due to conformational changes or electronic effects. It is worth noting that the replacement of fluorine with chlorine drastically decreased the activity, which may be due to the lower electronegativity of the Cl atom [23].

The third pair reveals the significance of the CF_3_ group, which is an often introduced substituent. In this case, fluorine was used for tuning the selectivity to dopamine D2/D3 receptors [24]. Fluorine has different properties depending on whether it is attached to the aliphatic or aromatic part of a compound. 

In the last example, the fluorine drastically changed the ligand conformation due to changes in electron density fluctuations along the aliphatic chain [25].

The fluorine substitutions discussed above were employed as a standard strategy to increase biological activity, without a defined underlying rationale. Very often the place of fluorine substitution is dictated by synthetic considerations, as fluorination in many cases requires difficult and time-consuming synthetic procedures.

### 3.3. MMP Network

To further extend SAR analysis of FSAR sets, a newly designed variant of an MMP network with multiple information layers was generated. In a first step, 937 of the 1554 target-based FSAR sets were combined on the basis of shared non-F ligands into multi-target FSAR sets, as illustrated in Figure 3. The remaining 617 FSAR sets represented single-target sets. The 937 overlapping FSAR sets yielded 349 multi-target sets comprising two to 13 target-based sets. Each of the 349 multi-target sets was based upon a non-F compound with activity against multiple GPCRs and contained one to 37 fluorinated analogues with variable target annotations. As a consequence, the potency effects for different targets often varied. Therefore, the potency effects of the original target-based FSAR sets were compared and a final potency effect was assigned, as specified in Materials and Methods. 

**Figure 5:**
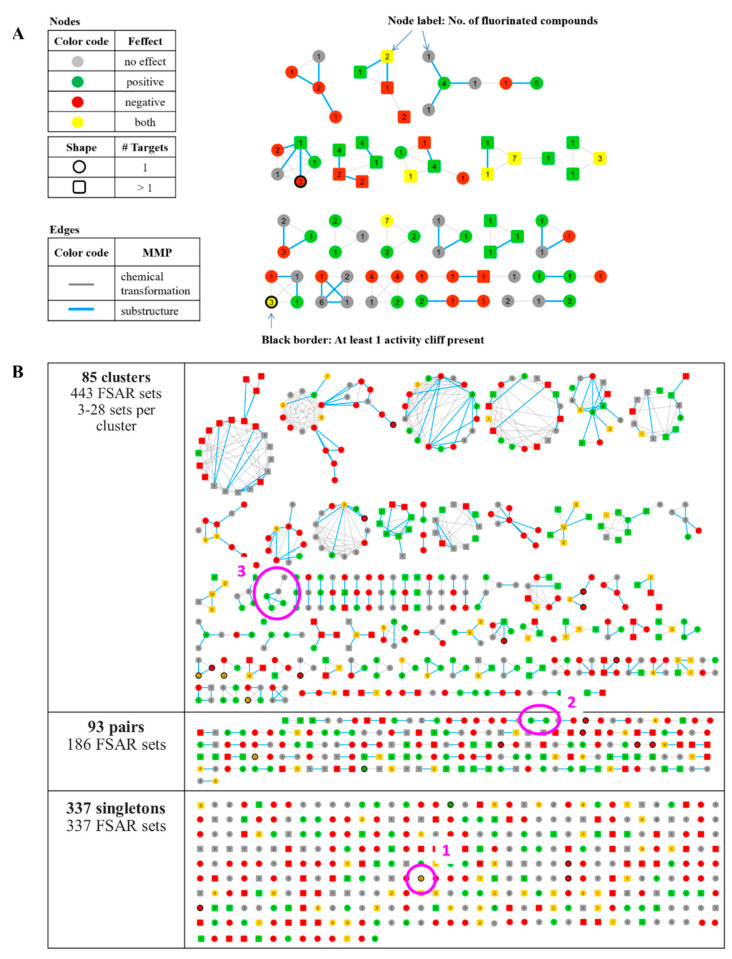
**MMP networks.** (**A**) The schematic depicts different information layers contained in the network. Nodes (non-F ligand and the corresponding FSAR set) and node shape account for single- (circle) or multi-target (square) activity. The color of the node represents the potency effect (green: positive, red: negative, yellow: both, and grey: no effect). Edges between nodes are drawn if the corresponding compounds form MMPs (grey). If one of the nodes is a substructure of the other, the edge is colored in blue. A thick black node border indicates the presence of at least one AC in the FSAR set. (**B**) The network for all 966 FSAR sets is shown. At the top, 85 complex clusters are formed between 3 to 28 FSAR sets. In the middle, FSAR set pairs, formed by two FSAR sets, and at the bottom 337 FSAR sets with no structural neighbors are shown. Exemplary clusters (1, 2, and 3) are encircled in pink and shown in detail in Figure 6 (A, B, and C, respectively).

The resulting 966 FSAR sets (617 single- plus 349 multi-target sets) were used to generate the MMP network, in which single-target sets were represented as circles and multi-target sets as squares. In the network, FSAR sets formed distinct clusters with structurally similar compounds. Figure 5A illustrates the MMP network structure with its different information layers and Figure 5B shows the network for the 966 FSAR. It is divided into three parts depending on the formation of clusters. At the top, 85 clusters formed by 443 FSAR sets are shown. In these clusters, at least three FSAR sets were connected and hence structurally analogous. The maximum number of FSAR sets in one cluster was 28. In the middle, 93 pairs derived from 186 FSAR sets are shown. A pair was formed by two structurally analogous FSAR sets. At the bottom, the 337 FSAR sets that did not form any MMPs with another set (singletons) are shown. Around 65% of the FSAR sets had at least one structurally analogous FSAR set (clusters and pairs in the network). A systematic analysis of structurally similar FSAR sets might confirm already observed SAR trends formed by adding or deleting fluorine atoms or can complement SAR information for individual FSAR sets. From each subnetwork—singletons, pairs, and clusters—three examples are analysed in more detail below.

**Figure 6:**
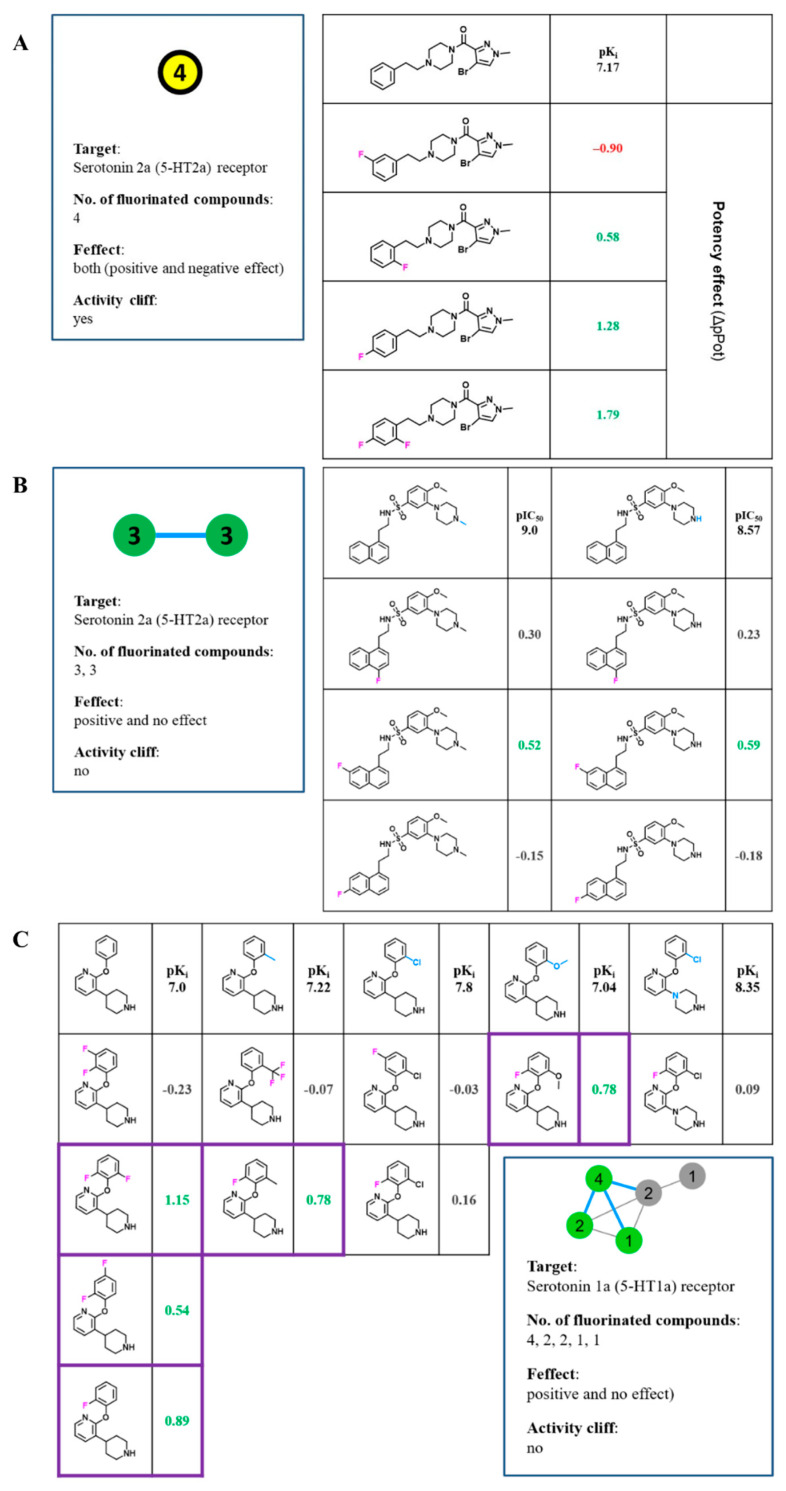
**Exemplary FSAR sets from the network.** (**A**) The FSAR set consists of one non-F compound and four fluorinated analogues annotated against the serotonin 2a (5-HT2a) receptor. The fluorinated compounds show negative and positive potency effects and the set contains an AC depending on the position of the fluorine atom. (**B**) The pair of FSAR sets comprising a non-F compound and three fluorinated analogues in each case with activity against the 5-HT2a receptor. The FSAR sets differ only in a methyl group and show fluorine substitutions at identical sites with the same positive potency effects. (**C**) A more complex cluster is shown that combines five FSAR sets active against the serotonin 1a (5-HT1a) receptor. The number of fluorinated compounds differs from one to four analogues per set. Three of the five sets have a positive and two no potency effect, although the recurrent *ortho* position of the fluorine atom (highlighted in purple) always shows a significant positive effect.

In the first series of 5-HT2a ligands (Figure 6A), the fluorination of an aromatic ring significantly influenced the affinity and selectivity for the 5-HT2a receptor (5-HT2c was an off-target) [26]. While a single substitution in *para* and *ortho* positions improved potency (ΔpK*_i_* = 0.58 and 1.28, respectively), an introduction of fluorine at both positions resulted in AC (ΔpK*_i_* = 1.79), whereas the meta substituted derivative exhibited a strong negative effect (ΔpK*_i_* = −0.90).

The next FSAR example in Figure 6B comprises sulfonamide 5-HT2a ligands with a difference in the piperazine fragment, in which fluorinated analogues showed the same trend of affinity changes in both sets. The substitution of fluorine at the 4- and 7-positions of the naphthalene moiety led to a slight increase in affinity (ΔpK*_i_* in the range of 0.23−0.59, respectively), whereas substitution at the 6-position caused a slight decrease (ΔpK*_i_* ~ −0.2) [27].

The last example (Figure 6C) shows MMPs with the largest combination of fluorinated analogues [28]. The analysis revealed that F atoms could serve as hydrogen bioisosteres, but at the same time, could dramatically change the selectivity of the analogues. While the set did not contain ACs, fluorination at *ortho* and *para* positions or their combination modulated the affinity. The compound with the highest ΔpK*_i_* values (1.15) had two fluorine atoms in the *ortho* positions. When fluorine was found at the *meta* position the affinity decreased, additionally, the OCF_3_ group in the *ortho* position was also unfavorable, which was likely due to the steric effect or conformational changes [28].

### 3.4. SAR Rules

The example discussed above revealed frequent potency effects as a consequence of fluorination of closely related compounds. To derive the rules of thumb for fluorination of aminergic GPCR receptor ligands, a statistical analysis of the sites of fluorine substitution and the effects on biological activity was performed. The results indicated that the fluorination of the aromatic ring showed no clear trend towards a positive or negative effect on affinity (556/619 positive/negative fluorinated derivatives, respectively), as reported in Table 1.

However, analysis of the fluorine substitution site in the aromatic ring showed that fluorine in the ortho position was twice as likely to have a positive effect than a negative one; for the meta and para fluoro derivatives, no trend was observed (Table 2). Surprisingly, fluorine found in the aliphatic moiety of compounds had five times more often a negative influence on biological activity (Table 1). It is worth noting that among 16 aliphatic fluorination-dependent ACs, 15 were negative and only one positive.

## 4. Conclusions

Herein, we have systematically explored the effects of fluorination on aminergic GPCR ligands. Nearly 1200 non-F ligands were identified for 35 aminergic GPCR targets that had at least one fluorinated analogue. Detailed analysis of newly derived FSAR sets revealed position-dependent effects of compound and identified a limited number of ACs. An overall favourable site for a positive potency effect of fluorination was the ortho position in an aromatic ring. Fluorination of aliphatic fragments more often led to a decrease in biological activity. Although fluorine is generally regarded as a bioisostere of the hydrogen atom, single fluorine substitutions can lead to significant changes in selectivity of bioactive compounds and also change of its biological function (e.g., from inverse agonist to antagonist). 

The results presented herein provide insights into the role of fluorine substitutions in ligands of aminergic receptors of class A GPCRs and can be applied in rational drug design to guide modification improving pharmacodynamics.

## Figures and Tables

**Table 1:** The number of fluorinated compounds in each ΔpPot range.

The Type of Fluorine	Fluorine Attached to an Aromatic Carbon	Fluorine Attached to Aliphatic Carbon
no. of positive fluorinated compounds ΔpPot > 0.3	556	21
no. of negative fluorinated compounds ΔpPot < −0.3	619	97
no. of positive AC	16	1
no. of negative AC	22	15

**Table 2:** The number of fluorinated aromatic ring site of compounds in each ΔpPot range.

The Site of Fluorine Substitution	No. of Positive Fluorinated CPDs ΔpPot > 0.3	No. of Negative Fluorinated CPDs ΔpPot < −0.3
ortho	54	21
meta	55	50
para	125	144

## Data Availability

The data were obtained from the ChEMBL database (https://www.ebi.ac.uk/chembl/, (accessed on 10 October 2021)).

## Supplementary Materials

The following are available online at https://www.mdpi.com/article/10.3390/biom11111647/s1, Table S1: The list of 33 GPCRs, which FSAR sets were identified in the ChEMBL database.
