# Supplementary material for: Data-Driven Analysis of Fluorination of Ligands of Aminergic G Protein Coupled Receptors
Source: Biomolecules. 2021 Nov 8;11(11):1647. doi: 10.3390/biom11111647 (PMC8615825; doi:10.3390/biom11111647)
Supplement: Supplementary file 1 [file biomolecules-11-01647-s001.zip › biomolecules-1441070-supplementary.pdf]

Supplementary Materials

Data-Driven Analysis of Fluorination of Ligands of Aminergic G Protein Coupled Receptors

Wojciech Pietruś [1,2], Rafał Kurczab [1,*], Dagmar Stumpfe [2], Andrzej J. Bojarski [1] and Jürgen Bajorath [2,*]

1 Department of Medicinal Chemistry, Maj Institute of Pharmacology, Polish Academy of Sciences, Smetna 12, 31-343 Krakow, Poland; pietrus@if-pan.krakow.pl (W.P.); bojarski@if-pan.krakow.pl (A.J.B.)
2 LIMES Program Unit Chemical Biology and Medicinal Chemistry, Department of Life Science Informatics, B-IT, Rheinische Friedrich-Wilhelms-Universität, Endenicher Allee 19c, D-53115 Bonn, Germany; stumpfe@bit.uni-bonn.de
* Correspondence: kurczab@if-pan.krakow.pl (R.K.); bajorath@bit.uni-bonn.de (J.B.); Tel.: +48-126-62-3301 (R.K.); +49-228-73-69100 (J.B.)

Table S1. The list of 33 GPCRs, which FSAR sets were identified in the ChEMBL database.

ChEMBL ID	Name	#FSAR sets	#Fluorinated CPDs
CHEMBL3371	Serotonin 6 (5-HT6) receptor	168	274
CHEMBL217	Dopamine D2 receptor	170	257
CHEMBL224	Serotonin 2a (5-HT2a) receptor	151	222
CHEMBL214	Serotonin 1a (5-HT1a) receptor	116	204
CHEMBL234	Dopamine D3 receptor	128	147
CHEMBL3155	Serotonin 7 (5-HT7) receptor	78	126
CHEMBL225	Serotonin 2c (5-HT2c) receptor	95	118
CHEMBL264	Histamine H3 receptor	73	112
CHEMBL245	Muscarinic acetylcholine receptor M3	52	71
CHEMBL1833	Serotonin 2b (5-HT2b) receptor	51	65
CHEMBL216	Muscarinic acetylcholine receptor M1	39	64
CHEMBL3759	Histamine H4 receptor	40	62
CHEMBL219	Dopamine D4 receptor	38	56
CHEMBL1983	Serotonin 1d (5-HT1d) receptor	20	47
CHEMBL229	Alpha-1a adrenergic receptor	26	46
CHEMBL1821	Muscarinic acetylcholine receptor M4	22	42
CHEMBL1898	Serotonin 1b (5-HT1b) receptor	15	34
CHEMBL231	Histamine H1 receptor	28	33
CHEMBL210	Beta-2 adrenergic receptor	23	31

CHEMBL246	Beta-3 adrenergic receptor	23	29
CHEMBL211	Muscarinic acetylcholine receptor M2	21	28
CHEMBL232	Alpha-1b adrenergic receptor	19	28
CHEMBL213	Beta-1 adrenergic receptor	20	27
CHEMBL223	Alpha-1d adrenergic receptor	15	19
CHEMBL1867	Alpha-2a adrenergic receptor	13	17
CHEMBL2035	Muscarinic acetylcholine receptor M5	15	16
CHEMBL2056	Dopamine D1 receptor	11	14
CHEMBL1941	Histamine H2 receptor	10	12
CHEMBL1916	Alpha-2c adrenergic receptor	10	11
CHEMBL1875	Serotonin 4 (5-HT4) receptor	8	9
CHEMBL1942	Alpha-2b adrenergic receptor	7	8
CHEMBL3426	Serotonin 5a (5-HT5a) receptor	1	3
CHEMBL2182	Serotonin 1e (5-HT1e) receptor	1	1
